# Sleep Disorders in Rett Syndrome and Rett-Related Disorders: A Narrative Review

**DOI:** 10.3389/fneur.2022.817195

**Published:** 2022-03-01

**Authors:** Giorgia Tascini, Giovanni Battista Dell'Isola, Elisabetta Mencaroni, Giuseppe Di Cara, Pasquale Striano, Alberto Verrotti

**Affiliations:** ^1^Department of Pediatrics, University of Perugia, Perugia, Italy; ^2^Pediatric Neurology and Muscular Diseases Unit, IRCCS “G. Gaslini” Institute, Genoa, Italy; ^3^Department of Neurosciences, Rehabilitation, Ophthalmology, Genetics, Maternal and Child Health, University of Genoa, Genoa, Italy

**Keywords:** Rett syndrome, Rett-related disorders, sleep disorders, MECP2, CDKL5, FOXG1

## Abstract

Rett Syndrome (RTT) is a rare and severe X-linked developmental brain disorder that occurs primarily in females, with a ratio of 1:10.000. *De novo* mutations in the Methyl-CpG Binding protein 2 (MECP2) gene on the long arm of X chromosome are responsible for more than 95% cases of classical Rett. In the remaining cases (atypical Rett), other genes are involved such as the cyclin-dependent kinase-like 5 (CDKL5) and the forkhead box G1 (FOXG1). Duplications of the MECP2 locus cause MECP2 duplication syndrome (MDS) which concerns about 1% of male patients with intellectual disability. Sleep disorders are common in individuals with intellectual disability, while the prevalence in children is between 16 and 42%. Over 80% of individuals affected by RTT show sleep problems, with a higher prevalence in the first 7 years of life and some degree of variability in correlation to age and genotype. Abnormalities in circadian rhythm and loss of glutamate homeostasis play a key role in the development of these disorders. Sleep disorders, epilepsy, gastrointestinal problems characterize CDKL5 Deficiency Disorder (CDD). Sleep impairment is an area of overlap between RTT and MECP2 duplication syndrome along with epilepsy, regression and others. Sleep dysfunction and epilepsy are deeply linked. Sleep deprivation could be an aggravating factor of epilepsy and anti-comitial therapy could interfere in sleep structure. Epilepsy prevalence in atypical Rett syndrome with severe clinical phenotype is higher than in classical Rett syndrome. However, RTT present a significant lifetime risk of epilepsy too. Sleep disturbances impact on child's development and patients' families and the evidence for its management is still limited. The aim of this review is to analyze pathophysiology, clinical features, the impact on other comorbidities and the management of sleep disorders in Rett syndrome and Rett-related syndrome.

## Introduction

In recent years, attention to sleep disorders has increased especially in people suffering from neurodevelopmental disabilities such as: Rett syndrome (RTT), Down syndrome, Fragile-X syndrome, Prader-Willi syndrome, Angelman syndrome, Tuberous Sclerosis and Autistic spectrum disorders (1). The prevalence of poor sleep appears greater in adults and children with intellectual disability (ID) (respectively 8.5–31% and 16–42%) compared to individuals with a typical development (1, 2). Several sleep profiles have been described in genetic syndromes associated with ID. According to Agar et al. the variability in the prevalence of various sleep disorders underlies different etiopathogenetic mechanisms. A greater awareness for appropriate risk stratification and targeted therapy is necessary to improve the quality of life of these patients and their families (3). The aim of this review is to analyze the pathophysiology, clinical features and impact of sleep disorders in RTT syndrome and RTT-related syndrome.

## Rett Syndrome and Rett-Related Disorders

RTT is a rare and severe X-linked developmental brain disorder, predominantly affecting females with a ratio of 1:10,000 (4, 5). RTT represents the second cause of intellectual disability in females after Down syndrome (6). First described by Andreas Rett in 1960's (7), RTT was only later characterized by Hagberg et al. in classical form ad atypical Rett (AR) (8, 9). Methyl-CpG-binding protein 2 (MECP2) gene mutations were first described in RTT, suggesting the monogenic origin of the disease (10, 11). To date, more than 800 *de novo* mutations of MECP2 gene are responsible for 95% cases of classical RTT. Rare familial cases of RTT have also been described. AR is associated in only 50–70% of cases with MECP2 mutations. Other genes involved in AR are cyclin-dependent kinase-like 5 (CDKL5) and forkhead box G1(FOXG1) (12). Clinical presentation and severity of the disease can be extremely variable (13–15). Males with MECP2 mutations range from fatal neonatal encephalopathy to classical RTT and psychiatric symptoms such as schizophrenia or bipolar disorder (16, 17). The classical RTT onset is between the 6th and 18th month in subjects with mute gravidic history and born with a normal delivery. A normal psychomotor development is usually described in first months of life (5, 18). However, there is a growing body of literature supporting the presence of alterations from the earliest stages of ontogeny. Early impairment of gross motor skills, alterations in general movements, fine movements of the face and arms, stereotypes of the hands and tremors have been described in the first months of life. Behavioral alterations, with a poor repertoire of communicative gestures and characteristics like autism disorder have also been reported (8, 18–21). According to the 2010 revised criteria (Table 1), regression is a necessary cornerstone for diagnosis along with 4 main criteria (5). Classical RTT evolution can be summarized in four stages. A first phase (6–18 months) featured by deceleration of the development. The second phase (1–4 years) is characterized by skill regression over weeks or months and autistic-like behavior. Clapping or wringing replace the child's purposeful movements. Ataxia and apraxia could also appear at this stage of the disease. Autonomic disorders are frequent and responsible for respiratory alterations such as hyperventilation or apnea. Sleep disorders, bruxism, tremors, inconsolable crying or screaming are extremely common. The third phase (2–10 years) is characterized by a certain recovery or motor stabilization, improved interaction and a reduction in irritability. However, seizures may occur. The fourth phase (>10 years) is characterized by a continuous and progressive deterioration of motility, parkinsonism, joint contractures, scoliosis and other orthopedic problems. Epilepsy may improve (15, 22). “Atypical or variant” Rett, is defined by the presence of many of the clinical characteristics of RTT, but not completely satisfied. A period of regression in addition to at least two of the four basic criteria and five of the 11 supporting criteria are necessary for AR diagnosis (Table 2). Among the atypical forms are described the preserved speech variant (Zappella variant) mainly caused by MECP2 mutations, the congenital variant (Rolando variant) closely related to mutations in the FOXG1 gene and the early seizures variant (Hanefeld variant) often related to the CDKL5 gene (5, 19). To date, several Rett syndrome databases are available, reporting the clinical and molecular characteristics of affected patients: International Rett Syndrome Association (ISRA), North American database, InterRett and Rett Networked Database (RND) (23).

**Table 1:** Classic RTT: diagnostic criteria.

**Main criteria**	
A period of regression followed by recovery or stabilization	1. Partial or complete loss of acquired purposeful hand skills.
	2. Partial or complete loss of acquired language skills
	3. Gait abnormalities: impaired or absence of ability.
	4. Stereotypic hand movements (hand wringing/squeezing, clapping/tapping, mouthing and washing/rubbing automatism)

*Modified from (5)*.

**Table 2:** Main from supportive criteria.

**Main criteria**	
A period of regression followed by recovery or stabilization	96–99%
1. Partial or complete loss of acquired purposeful hand skills	87–100%
2. Partial or complete loss of acquired language skills	87–99%
3. Gait abnormalities: impaired or absence of ability.	45–99%
4. Stereotypic hand movements (hand wringing/squeezing, clapping/tapping, mouthing, and washing/rubbing automatism)	68–100%
**Supportive criteria**	
1. Breathing dysrhythmias when awake	54–99%
2. Bruxism when awake	62%
3. Impaired sleep pattern	45–80%
4. Abnormal muscle tone	–
5. Peripheral vasomotor disturbances (small, cold hands and feet)	75–99%
6. Scoliosis or kyphosis	≥52%
7. Growth retardation or deceleration of head growth	54–80%
8. Small cold hands and feet	–
9. Inappropriate laughing or screaming spells	30%
10. Diminished response to pain	–
11. Intense eye communication	88%

*Percentages of features shown by patients, when available. Modified from (16)*.

### CDKL5-Related Disorder

Disruptions in the CDKL5 gene lead to a rare X-linked neurodevelopmental disorder, which in some cases meets the criteria for AR (Hanefeld variant) (24). This gene was first described in 1998 during a transcriptional mapping project and encodes for a novel protein kinase located on the X chromosome (25). Subsequently in 2003, CDKL5 mutations were associated with a phenotype characterized by severe intellectual disability and early seizures onset. In 2004, CDKL5 rearrangements were identified in four cases of females with RTT (26, 27). Affected patients may present typical dysmorphic features: large deep-set eyes, prominent forehead, anteverted nares, full lips and wide mouth (26, 28). The clinical features of CDKL5-related disorder include early epilepsy onset, global developmental delay, involvement of gross motor skills, abnormal muscle tone, sleep disorders, gastrointestinal problems and breathing irregularities (26, 28). Seizures represent an hallmark feature, ranging from infantile spasms to tonic, myoclonic and complex partial seizures (29, 30). Hanefeld variant presents at least one episode of epilepsy. According to Frullani et al. more than half of children with CDKL5 mutations do not learn to walk. 80.3% of females in the InterRett database and 85.72% of females in RND show stereotypes of the hands. Differences emerged regarding language acquisition. In the international CDKL5 Disorder Database 22.7% of patients reached the easiest level of communication. In the InterRett group 39.5% had early language acquisition while in the RND only 17.4% of females showed some degree of speech acquisition (23, 26, 31). According to Mangatt et al. 50.9% of patients reported difficulty in oral feeding. Over 2 years of age, more than half of patients were completely dependent on caregivers for both eating and drinking; gastrostomy or dijunostomy was required in 28.8% of the cases (28). Comorbidities associated with CDKL5-related disorder increase with age. Males are at greater risk of developing respiratory and sleep disorders. In a study by Fehr et al. 86 patients with CDKL5 mutations were compared with 920 patients with MECP2 mutations. Epilepsy, sleep abnormalities, gastrointestinal disorders (constipation, gastroesophageal reflux and air swallowing) were more frequent in patients with CDKL5 mutations than RTT, while scoliosis and respiratory problems had a lower prevalence (23, 26).

### FOXG1 Related-Disorder

Forkhead box G1 (FOXG1) mutation was first described in 2005 in a girl with microcephaly, global developmental delay and agenesis of the corpus callosum (32). Point mutations, frameshifts mutations, deletions and duplications of FOXG1 underlie FOXG1-related encephalopathy including the congenital variant of RTT (33–35). FOXG1 deletions or intragenic mutations present postnatal microcephaly, neurodevelopmental delay from early childhood, visual deficits and poor feeding. Language is often minimal or absent. Hypotonia, hyperkinetic movement disorders and lack of gait acquisition may occure (34, 36). Hallmark features are generalized choreoathetosis, oral/facial dyskinesias, feeding difficulties and sleep disorders arising within the first year of life. Contrary to RTT, FOXG1 mutations show non-medial stereotypies, occurring while stroking, grasping and pedaling (36). Seizure onset by the second or third year of life is typical in FOXG1 deletions. Whereas, seizures occurs from the third to seventh month of life in FOXG1 duplications. Seizure ranges from generalized tonic-clonic to focal or myoclonic seizures. Infantile spams are more common in infants with FOXG1 duplication and are generally responsive to ACTH (37, 38). Seizure prevalence in FOXG1 deletion is 68–87% slightly higher than in classical RTT (39). Brain abnormalities include hypogenesis of the corpus callosum, delayed myelination and fronto-temporal atrophy. Hypoplastic hippocampus has also been described in a patient carrying a FOXG1 missense mutation (c.569T>A, p.Ile190Asn) (36). In the few described cases of FOXG1 duplication, the clinical picture is more attenuated. Affected children have an adequate head circumference and cerebral abnormalities are rarer and aspecific. Walking is achieved by 2 years of age. Patients show a global retardation, with autistic behavior and language disorders (38, 39).

### MECP2 Duplications Syndrome

MECP2 duplication or more rare triplications concerns about 1% of male patients with ID (5). MECP2 triplication is most associated with death in early childhood. According to a review by El Chehadeh et al. carriers mothers were usually asymptomatic and presented a skewed X chromosome inactivation pattern (40). MECP2 duplication syndrome (MDP) is related to the overexpression of MECP2 gene and can overlap with RTT. Clinical features of MDP are learning impairment, developmental regression, hypotonia and progressive spasticity, epilepsy, hand stereotypes, ataxia, small and cold feet, sleep disorders and dysrhythmias of breathing during wakefulness (17, 41, 42). Typical dysmorphisms, which become more pronounced with age, are thick lower lip, large pinnae, midface hypoplasia, narrow and prominent nasal bridge, thick hair, livedo of the limbs and vasomotor disturbances. Approximately 89% of patients present stereotypic movements. Stereotypies appear at around 3 years of age, without necessarily interfering purposeful hand use. Whereas, stereotypies in RTT replace intentional hand movements around 2 years of age. Neurodevelopmental delay associated with hypotonia were found to be constant, and ~21% of subjects were unable to walk. Another characteristic feature is the lack of expressive language. A singular habitus with knees in flexion and pseudo ataxia was described in patients able to maintain an upright position, due to muscle weakness and spasticity (16, 43, 44). Up to 60% of patients develop epilepsy with a later onset than RTT at around 8 years old (43, 45). Seizures are generally poor responsive to therapy and at least half of cases present the criteria of Lennox-Gastaut syndrome (16, 43, 45). Approximately 70–75% of patients develops immunodeficiencies and recurrent respiratory infections due to hypoventilation and other comorbidity. Severe forms usually require frequent hospitalizations and ventilatory support from an early age (44, 46). Urinary tract infections, meningitis or sepsis have also been described. According to Bauer et al., patients more prone to infections show an IgA/IgG deficiency (42, 47). There are several cases described in the literature with abnormal breathing pattern (grunting, aerophagia and snoring). Belligni et al. described a case of congenital hypoventilation syndrome in a patient with MDS. A French survey conducted on 59 patients showed a prevalence of OSA of 33%. Therefore, altered sleep breathing and nocturnal hypoventilation should be considered in this condition and are worthy of further investigation (43, 46, 48). Approximately 25% of patients with MECP2 duplication syndrome often die of respiratory infections and pulmonary hypertension even before the age of 25 years (16, 43).

### MECP2, CDKL5, and FOXG1 Genes and Their Protein

RTT, as a monogenic disease, has paved the way for many cellular and transgenic animal models: MECP2 mutant models (knock-in mouse models with specific MECP2 mutations), MECP2 deficient models (MECP2 constitutive knockout mice or brain region/cell type-specific deletion of MECP2). To date, MECP2 deficient cultured neurons have been employed in human RTT cellular models systems (49). MECP2 gene is located on the long arm of the X chromosome (Xq28) (12). Four coding exons and three introns compose the gene (MECP2 in humans and MECP2 in mice). Moreover, three polyadenylation sites in its 3'UTR are recognized, resulting in different mRNA transcripts. MECP2 protein belongs to the Methyl-CpG-binding protein family, consisting of 11 members. Five main domains compose the protein: N-terminal domain, Methyl-CpG binding domain (MBD), intervening domain, transcription repression domain (TRD) and C-terminal domain. MECP2 is express in neurons, neuronal stem cells, astrocytes and oligodendrocytes (12), both in the nucleus and cytoplasm. It plays an important role in neuronal maturation and plasticity and its deficiency leads to important cellular abnormalities. Expressed at low levels in the prenatal period, it peaks during neuronal maturation and synaptogenesis (50). The common splice variants of the protein (MECP2E1 and MECP2E2), which differ in N-terminal domains, originate from the two translation start sites at exon 1 and 2. Only mutations in exon 1 are involved in RTT. MECP2E1 is the main isoform of the central nervous system and arises from the coding sequences of exon 1, 3, and 4. It is homogeneously expressed in the cortex, thalamus, hippocampus, brain stem and cerebellum, during the early stages of brain development. MECP2E2 is highly expressed at placenta, liver, skeletal muscles and prostate gland and results from exons 2, 3, and 4 (51, 52). It is expressed in a later stage and presents a peculiar pattern in different brain regions (49, 53, 54). MECP2 binding methylated DNA sites acts as a transcriptional modulator (55). In addition, MECP2 inhibits gene regulation bounding to mSIN3A and NCoR1/SMRT co-repressor complexes. It is involved, in chromatin compaction and in the modulation of RNA splicing (49, 50, 55, 56). MECP2 mutations are responsible for 95% of RTT cases and more than 50% of AR. Forty-seven percentage of all mutations are due to eight variants: four affecting the TRD (R255X, R270X, R306C, and R294X), three the MBD (R106W, R133C, and T158M) and one the intervening domain (R168X) (50, 57). C-terminal deletions are associated with milder phenotypes while T158M mutation is responsible for severe forms (12, 49, 58, 59). The R168X mutation is the second most frequent mutation (60). TRD mutations result in the loss of MECP2 interaction with NCor/SMRT co-repressor (60). C-terminal Domain seems to intervene in histone binding and transcriptional activation of brain-derived neurotrophic factor through the S421 serine residue (49, 61). Moreover, CDKL5 and FOXG1 seem to converge on MECP2, which is involved in forebrain differentiation (62). *De novo* mutations affect more frequently the paternal germ line, with a greater involvement of females. Healthy carriers or mildly affected female due to X chromosome inactivation can pass the mutation to their offspring explaining the rare cases of familial forms. Gonadal mosaicism can also underlie familiar inheritance of RTT (63). Takahashi et al. described a 12-year-old girl with X chromosome mosaic karyotype (46, XX/47, XXX) fulfilling the diagnostic criteria for the preserved speech variant. The authors showed how the nature of supernumerary X chromosome, XCI status and the mosaicism interfere with phenotype variability (64). CDKL5 gene, located on Xp22, is 228 Kb in length and consists of 27 exons. It encodes for a serine/threonine protein kinase. One of CDKL5 isoforms is predominantly expressed in the cerebral cortex, cerebellum, hippocampus, striatum and brainstem as well as in testes and thymus (25, 65). CDKL5 is localized in both the nucleus and cytoplasm and its location is constantly changing and linked to the cell cycle. CDKL5 is poorly expressed in the embryonic cortex and increases in the early postnatal stage and in adult. It is hypothesized to play a key role in dendritic spine morphology, neuronal cell death and dendritic architecture (12, 65, 66). The protein shares similarities with CDK and mitogen-activated protein kinases. It is characterized by a catalytic domain, consisting of the 12-subdomain structure of Ser/Thr kinases. Between subdomains VII and VIII, there is an activation loop whose autophosphorylation results in the activation of the catalytic function of CDKL5. Two nuclear localization signal sequences and a nuclear export signal are contained in the regulatory domain, which is in the C-terminus. Studies *in vitro* showed how these signal sequences regulate the intracellular localization of CDKL5 (25, 67, 68). Mutations leading to loss of protein function are thought to be responsible for the neurological alterations that characterize the CDKL5 disorder, but to date little is known about CDKL5 substrates. Katayama et al. described recent discoveries of the phosphorylation of CDKL5 targets and the mechanism of CDKL5 phosphorylation/dephosphorylation, stressing how crucial this is for the development of new drugs (25, 69, 70). In 2005, Mari et al. suggested a common signaling pathway between CDKL5 and MECP2, showing that the MECP2 gene product was phosphorylated by CDKL5 (71). However, MECP2 is phosphorylated by CDKL5 only moderately and some authors claim that CDLK5 does not directly phosphorylate MECP2 (69, 72). It addition, CDKL5 expression could be suppressed by MECP2 (73). High levels of Glutamate D1 receptor (GluD1) were found in induced pluripotent stem cells with both MECP2 and CDKL5 mutations (74). Further research into the interaction of these two proteins and their molecular mechanisms is needed. From 2004 many mutations in the CDKL5 have been described: point mutations, frame shift, missense and nonsense mutations and larger rearrangements (19). Missense mutations are predominantly located in the region coding for the catalytic domain and result in a loss of enzyme activity, which is responsible for the onset of the disease. Instead, nonsense mutations may lead to an altered localization of CDKL5, due to the lack of the regulatory domain (70). FOXG1 is located on chromosome 14q12 and it is 4,890 bp in length. FOXG1 encodes for a brain-specific transcriptional repressor involved in telencephalon and visual structures development. It is expressed from the early telencephalic neuroepithelial progenitors to adult period, leading the spatial subdivision (telencephalic dorsoventral and mediolateral compartments). It regulates the forming of the corpus callosum, cortical lamination and dendritogenesis. FOXG1 is involved in neuronal proliferation and differentiation. Its constitutive suppression allows the development of Cajal-Retzius cells from cortical progenitors (39, 75–78). Adequate levels of the protein are critical for the balance of inhibitory/excitatory neurons. As for MECP2 and CDKL5 mutations, high levels of GluD1 have been demonstrated in FOXG1 mutations resulting in an imbalance toward inhibitory synapses. The imbalance in favor of the inhibitory stimulus found in FOXG1, CDKL5, and MECP2-related disorders could be related to the clinical overlap of these syndromes (74, 79). Boggio et al. conducted a study aimed at investigating the role of FOXG1 in the visual system. Using a mouse model, the report showed a severe defect in visual acuity in FOXG1^+/cre^ mice compared to wild-type littermates, as an abnormal organization of excitatory/inhibitory circuits in the visual cortex, in the absence of retinal alterations. Visual dysfunction emerged in all FOXG1-mutated patients, confirming how haploinsufficiency of FOXG1 is involved in a deficit of the visual cortical function (80). In the last 15 years, more than 100 FOXG1 variants have been described, mostly *de novo* mutations (39). Mutations can affect the whole length of the gene but there are two mutations of particular interest in the N-domain: c.256dupC and c.460dupG. These two loci have repeated sequences, seven cytosines and seven guanines, respectively, which are the subject of replication errors (36, 38, 81). Mutation in the N-terminal leads to severe phenotypes, while milder phenotypes correlate with mutation at the C-terminal and forkhead binding domain (38, 39, 82). In recent years, thanks to the use of gene panels, whole exome sequencing (WES) and whole genome sequencing (WGS), it has been possible to obtain new genetic diagnoses with a considerable increase in the number of causative genes of RTT-like phenotypes. With NGS more than 80 genes have been correlated with RTT-like phenotypes and atypical RTT, although some of them are associated with known syndromes such as: Angelman syndrome (UBE3A gene), Kleefstra sydnrome (EHMT1), Pitt-Hopkins syndrome and others. Some genes are more represented than others, such as STXBP1, TCF4, SCN2A, WDR45, and MEF2C (52). There are heterozygous *de novo* germline mutations, with autosomal dominant, autosomal, or X-linked transmission models. Moreover, pathogenic variants of GRN1, K1F1A and CACNA1A should be considered in Rett-like patients (83, 84).

## Sleep Disorders in Rett Syndrome and Rett-Related Syndrome

RTT, as a progressive and severe disorder, is characterized by multisystem comorbidities, that evolve with age and require a multidisciplinary approach. It is important improving the quality of life of patients and their families, considering that as has been shown by a recent report based on a large North American longitudinal cohort, patients with RTT have life expectancy well into the fifth- sixth decade of life (85–87). In the context of such a complex syndrome with health and behavioral problems (autonomic dysregulation, motor impairment, epilepsy, swallowing dysfunction, gastrointestinal and orthopedic comorbidities) sleep problems are extremely frequent and relevant (85, 88). Being a physiologic behavior, sleep disturbances affect many patients with intellectual disability and despite a certain scarcity of research sleep disruption is reported in over 80% of individuals affected by RTT and the impaired sleep pattern is part of the diagnostic criteria (15, 89). In CDKL5 disorder, sleep abnormalities together with epilepsy and gastrointestinal problems have a higher prevalence than in classical RTT and increase with age. FOXG1 related syndrome has a sleep disturbance prevalence of about 64.3–72.7% compared to 67% in MECP2 mutation (26, 28, 39, 81). More studies are needed to clarify the pathophysiological mechanisms underlying sleep disorders. Nevertheless, highly fragmentated sleep and circadian rhythm alterations have been found in animal models (mutated models of cynomolgus monkeys, Drosophila, and mice). Evidence showed how MECP2, but also CDKL5 and FOXG1, might be implicated in the proper functioning of specific brain areas and neurotransmitters involved in the sleep-wake cycle (39, 90, 91). Electrical brain activity (EEG) that could be recorded both in patients and animal models, is a useful tool for studying abnormalities in brain functioning. In particular polysomnography can investigate abnormalities associated with sleep disturbances and epileptiform activity, which in RTT patients may be more frequently recorded during sleep than during wakefulness (19, 62). Boban et al. conducted a study, aimed at assessing the management and impact of sleep disorders in a group of 364 families with a child with RTT, registered in the International RTT phenotype database. Of the enrolled patients, 274 were not taking therapy, 7.7% were on melatonin, 3.9% on clonidine, 3.3 on trazodone and 4.1 on other therapies (clonazepam, chloral hydrate, diazepam, oxazepam, antiepileptics, hypnotics e.g., zolpidem, and others). Sleep hygiene strategies, however, remained the first-line treatment and were adopted by 2/3 of the families, with benefit (89). Although drug studies on RTT syndrome have increased considerably in recent years, as also reported by Gomathi et. al, with progress being made in both clinical and non-clinical studies, the evidence for the management of sleep disorders is still limited, and melatonin, GABA agonists and dopamine agonists remain the most widely used drugs (15, 89, 92).

### Clinical Features of Sleep Disorders in Rett Syndrome and Rett-Related Disorders

Sleep-associated problems are a prevalent neurological comorbidity of RTT, from the earliest descriptive literature, sleep dysfunction emerged (9, 93–95). Piazza et al. in (96) analyzed 20 girls with RTT. They showed that the patients' total sleep duration was longer than that of healthy peers. In addition, there was a reduction in night sleep inversely proportional to age, while daytime sleep appeared increase and positively correlated with age. Altered sleep/wake patterns seemed to worsen over time and delayed onset of sleep, night waking, and naps were also observed (96, 97). Indeed, sleep disorders reported by patients include dysregulation of the sleep/wake cycle, difficulty falling asleep or staying asleep, night laughter or screaming and frequent awakenings, inconsolable crying, sleep terrors, talking and nocturnal seizures (1). A subsequent study of a sub-set of the Australian population-based data confirmed the finding of increased sleep onset latency, nocturnal awakenings, and daytime naps, but the mean total sleep time remain quite constant, without a progressive reduction in total sleep as the condition progressed (93, 97, 98). Young et al. in (98) investigated sleep problems in a large sample of 237 cases from the Australian Rett syndrome database (ARSD), which provides information on the functional abilities, medical conditions, genotype, and sleep characteristics of the patients included. Patient caregivers were asked to complete a questionnaire at 3 separate times (2000, 2002 and in 2004). Sleep disorders were found in 80–94% of cases, consistent with subsequent studies, which included polysomnographic studies, and the most frequent were naps during the day, laughter during the night, night screaming, bruxism, and seizures (98). The data showed some variability in relation to age, with night laughter seeming to decrease over time in contrast to daytime naps (98). Although laughter can have positive effects on health, it can also be secondary to organic lesions of the central nervous system or brain dysfunction, and laughter can be a manifestation of seizures of the frontal and temporal lobes, hypothalamus, and cingulate cortex (99, 100). Young et al. report a prevalence of 58.9% of nocturnal laughter, a high prevalence of 83% has also been confirmed in an early US survey and Boban et al. reported night laughing for 58% of individuals, possibly due to dysfunction of the cortices involved (98, 101). The prevalence and elements of sleep disorders were further detailed by Boban et al. in (95), in 364/461 RTT and MECP2 mutation patients registered in the international Rett syndrome phenotype database (InterRett) (95). Among the specific sleep problems (difficulty falling asleep, night laughing, night screaming, seizures at night, teeth grinding, night waking, daytime napping, and difficulty waking) the highest prevalence was found for nocturnal awakenings and 48.3% of the enrolled individuals woke up more frequently at night, in agreement with previous studies (95, 97). Patients were also found to be particularly affected by delayed sleep onset and night laughing (95, 98, 101). Furthermore, while previous literature has shown a reduction in some problems with age, such as nocturnal laughter, in the international study by Boban et al., night laughing and difficulty falling asleep were also found to be higher in the group of patients under 7 years of age, while there was a progressive increase in nocturnal awakenings and the score of total sleep disturbance scale and subscale used were higher in this group (95, 97, 98). A recent prospective pilot study, using sleeping questionnaire for children with neurological and other complex disease (SNAKE) and Rett Syndrome Behavior Questionnaire (RSBQ), evaluated the quality of sleep and the relation between behavioral disorders and sleep quality, in a sample of members of the Elternhife fur Kinder mit Rett Syndrome in Deutschalnd e.V. The patients were divided into four groups according to age: group 1 included patients from 0 to 6 years, group 2 patients from 7 to 12 years, group 3 patients from 13 to 18 years and finally group 4 included patients over 18 years. Sleep disturbances in this study appeared less severe than previously reported results and sleep quality was described as good or very good in 60% in each group, probably also in relation to the subjective assessment provided by parents or caregivers (15). Furthermore, an improving trend with increasing age is described in accordance with the high prevalence of sleep disturbances shown in patients younger than 7 years and with Wong et al. who explored the relationship between age, mutation type and medicaments on sleep disruption (95, 97). Sleep improvement with age seems to occur in only a proportion of individuals and it is difficult to correlate sleep disturbances to a single trigger (97). The mutation type as well as other comorbidities are surely involved in the evolution of the disorder. The decrease of epileptic activity with age in classical Rett could also justify the improvement of sleep pattern in older patients. However, in the study by Level et al. sleep quality was also worse in the oldest patients. The worsening of general clinical conditions due to the aggravation of orthopedic problems such as scoliosis, motor deterioration in association with other comorbidities, as well as polytherapy may contribute to this finding (15). Halbach et al. also showed a deterioration in patients over the age of 30 and confirmed a reduction in sleep-related problems between the ages of 16 and 20 (102). Epilepsy has a high prevalence in RTT syndrome, from 48% as shown by Glaze et al. to 94% reported in other studies (103–106). Frequent seizures seem to be associated with poor sleep, as well as interfering with sleep architecture (95, 107). In MECP2 positive patients, epilepsy usually begins in stages II and III of the disease, between 7 and 12 years of age and an early onset seems to be associated with a worsening evolution toward forms that are poorly responsive to therapy, epileptic state. At the same time epilepsy may be over diagnosed in patients with RTT syndrome as also reported by some authors: only one-third of parent-reported seizure behaviors were found to have epileptiform abnormalities on EEG (12, 19, 104). After 3 years of age, the EEG of a person with RTT is characteristically abnormal, also during sleep (slowing and sporadic epileptiform discharges, spike- and-slow-wave activity). Treatment is only prescribed by experts, if there is a clear diagnosis of seizures and it is important to use long-lasting video EEG in situations of uncertain significance (103). Epilepsy affects the quality of life, patients appear more tired and less responsive throughout the day, and the sedative effects of anti-epileptic therapies may also contribute to sleep disorders of older individuals (95, 107–110). Insomnia is characterized by a high prevalence in subjects with neurodevelopmental disabilities but is not associated with a specific genetic risk. Probably, in this type of subject, intellectual disability and the environment have a strong influence. Associated comorbidities such as epilepsy and hence anti-epileptic therapies, along with differences in sensory processing, mobility problems and the reduced influence of zeitgebers, that drag the sleep-cycle, builds an additional risk factor (1, 111). For the study of sleep, the use of actigraphy has also proved to be reliable, allowing recording while the patient is in his or her usual environment and over a long period of time. However, in RTT patients with severe orthopedic involvement, the use of actigraphy may not be as reliable. Merbler et al. conducted a study of 13 patients comparing the data obtained from the actigraphy with a sleep diary completed by the parents and the Child Sleep Habit's questionnaire (CSHQ). Fifty percentage of the participants showed a total sleep time below the recommended ranges according to the parents' reports, onset latency was resulted appropriated, and the sleep efficiency was found to be associated with seizures (88). In relation to genotype, daytime napping is frequently in p.T158M, p.R270X and p.R255X mutations. P.R270X, p.R255X are nonsense mutations and are associated with a more severe phenotype. Nevertheless, sleep problems are frequent in individuals carrying mutations related to mild phenotypes, such as p.R294X, p.R306C and C-terminal mutations. Night laughter and sleep disturbances are found in almost all cases with large MECP2 mutations. Finally, Boban et al. reported that sleep onset and sleep maintenance is more pronounced in younger patients and those with the p.Arg294 mutation (95). Breathing disorders during wakefulness, such as hyperventilation phases followed by "respiratory arrest”, or air swallowing have been well-described in the literature and in recent years more attention has been paid to sleep-disordered breathing (SBD) (112, 113). Several studies have reported a high incidence of obstructive sleep apnea as well as reduced sleep efficiency (98, 114). Episodes of hyperventilation and subsequent apnea have been described in girls with RTT, while other studies have shown episodes of hypoventilation, reduced tidal volume and hypoventilation with some variability between nights and between patients (115–117). It has been suggested that abnormalities in the breath center may be the cause. In contrast in a study of 30 patients by Marcus et al. no abnormalities of breathing during sleep were found, and this may be due to the lower degree of severity of breathing abnormalities during sleep (112, 118). As a result, polysomnography is a valuable tool that should be part of the routine assessment of children with RTT (119). Sleep disturbances, specifically difficulty initiating and maintaining sleep, are an area of overlap between MECP2 duplication syndrome and RTT, although less represented in the former (16). Comparing CDKL5 disorder with RTT, the evidence shows that sleep disorders, epilepsy and gastrointestinal problems have a higher prevalence than spinal abnormalities and respiratory problems. Overall, patients with CDKL5 mutations show a progressive worsening of comorbidities over a lifetime. Males seem to be more affected by respiratory problems and sleep disorders (26, 28). A study, based on a large amount of data from the International CDKL5 Disorder Database (ICDD), found that 86.5% (122/141) had experienced sleep disturbances and night waking were the most frequent consistent with a French study, in which more than half of the patients manifested night waking and screaming spells (120). In addition, difficulties of initiating and maintaining sleep and daytime naps are reported in other studies by parents of children with CDKL5 mutations (121). Epilepsy in these patients contributes to an increase total sleep time and daytime sleep time. Disruption in the circadian rhythm, an altered balance between inhibitory and excitatory stimuli, intractable forms of epilepsy, gastroesophageal reflux and scoliosis may underlie sleep problems (28, 122, 123). No particular links between comorbidities and specific genotypes emerged but it would appear that late truncations after aa781 are protective against sleep disorders, autonomic breathing disorders and gastrointestinal problems (28). In FOXG1-related syndrome sleep disturbances seem to improve with growth. Difficulties in falling asleep associated with crying and irritable state, nocturnal awakenings and nocturnal laughter are the most represented (39, 81).

### Sleep Research in Both Animal Models and Humans

Sleep as well as circadian clock rhythmicity are needed by living species (124). Circadian clock in humans is found in a similar way in animals and locomotion's variation follow circadian rhythmicity (125). Thanks to the discovery of electrical brain activity, it was possible to investigate the sleep-wake cycle and using EEG technology, considered to be a neural biomarker and which can be recorded in animals and humans, translational research was made possible (90). As previously mentioned, sleep/wake cycle disorders are prevalent in RTT and MECP2, causative gene of the disease and highly expressed in the central circadian clock, suprachiasmatic nucleus (SCN) and photic stimulation leads to its phosphorylation (126, 127). Furthermore, there is a link between MECP2, other genes involved in chromatin remodeling and the core molecular clockwork, like MECP2, Ep300, and Jarid1a act on circadian clock genes (Per1 and Per 2), activating them transcriptionally in the mouse SCN. Quan li et al. in a RTT model, MECP2^−/*ymice*^, found an alteration of daily rhythms associated with sleep fragmentation. There was also a reduction in vasoactive intestinal peptide (VIP) due to a quantitative reduction of neurons located at the level of the SCN, with abnormal neural activity. The study reveals a disorganization of the entire circadian system, with interruption of the molecular clock at all levels, from the SCN to the peripheral organs. MECP2 mutant mice were also found to be particularly sensitive to circadian disruption. A possible role of MECP2 has emerged in the circadian system and a possible correlation with sleep problems observed in RTT patients (128, 129). Alteration in the circadian rhythms have been found also in the mouse model of CDKL5 deficiency disorders (130). Additionally, Liu et al. reported that FOXG1 is implicated in the development of the epithalamus, which is involved in the stress response and sleep-wake cycle in vertebrates, consistent with the clinical pattern (emotional disorders, sleep problems and choroid cysts) expressed by patients suffering from FOXG1-related disorders (131). Zhang et al. recently published a systematic review of sleep disturbances in RTT animal models, analyzing 13 studies in mutated models of Drosophila, cynomolgus monkeys and mouse, the latter the most used in current studies (90). Disruption in sleep continuity and efficacy are confirmed. Sleep abnormalities and an increased number of nocturnal awakenings were observed in all animal models (90, 132–134). Sleep duration was investigated by three studies, that showed an unchanged percentage duration of sleep during the day, but Johnston et al. reported a decrease of REM sleep cycles in mutants (135, 136). An altered respiratory pattern during sleep has been reported in three studies. In CDKL5 mutated mice sleep apneas appear more frequently in NREM than in REM sleep, as a progressive respiratory disruption characterizes MECP2 mutant mice too, in which apneas first appear in the waking state and then, after about 1–3 weeks, in sleep (90, 136–138). Sleep fragmentation emerged in mutant monkey and mouse models, in agreement with the frequent night waking also reported in RTT patients (129, 132). Overexpression of MECP2 in Drosophila appears to intervene in sleep continuation, studies in mice have shown long sleep onset latency and low sleep efficiency, suggesting a negative action of MECP2 on the regulation of sleep propensity (90, 103, 135). This could be traced back to the glutamatergic alteration found in MECP2-null mice, in which a biphasic trend in the expression of ionotropic glutamate N methyl-D-aspartate receptors (NMDARs) in the frontal cortex was shown, with an initial increased density in the young stage, progressively decreasing. Therefore, the initial overexpression of the excitatory stimulus in the initial stages could lead to a subsequent burn-out in older animals, in which, an imbalance in favor of the inhibitory stimulus is established (91, 139–141). In MECP2-null mice there is an altered sleep structure, an increase of cortical glutamate than in the wild type, a longer wake cycle predominate with an associated scarce quality of slow-wave sleep. This findings are consistent with studies in RTT patients which showed an impaired slow wave sleep pattern and a specular glutamate load (91, 135). The five-sleep stage was described first by Rechtschaffen and Kales, using polysomnography: REM sleep (REM, rapid eye movements) with an EEG similar to wakefulness, NREM sleep, during which there is a progressive transition from drowsiness to deep sleep and then a progressive increase in slow-wave activity (62). RTT patients show a reduction in the absolute duration of overnight SWS. However, studies show a reduced percentage of REM sleep compared to a longer time spent in SWS, especially in young girls aged between 2 and 5 years, with an electroencephalographic tracing characterized by increased delta activity during NREM sleep and an intense gamma activity in the occipital leads. This activity seems to decrease at later ages, supporting the hypothesis of an increased expression of glutamate receptors in younger affected subjects (62, 91, 142). Both ionotropic and metabotropic glutamate receptors appear to be involved in the pathogenic mechanism underlying RTT (91). While ionotropic receptors are overexpressed in RTT, mutations in MECP2 appear to reduce the transcription and epigenetically affect metabotropic receptors. Consistent with these results, the use of drugs targeting glutamate receptor has shown an improvement RTT phenotype. Both NMDAR antagonist and metabotropic receptors agonists were evaluated in preclinical studies (143–146). Further trials are warranted to better understand the potential use of this drugs in RTT patients.

### Sleep Disorders: Impact and Management

A multidisciplinary approach is necessary in patients with neurodevelopmental disorders, such as RTT, to identify the clinical features that have the greatest impact on the quality of life of the patient and their family (147). In fact, both can manifest symptoms in the psychological and physical spheres (148). Parents, who are dedicated to raising a disabled child, experience stress and may have an increased risk of cardiovascular disease and depression (149, 150). Sleep disturbances negatively affect children and their families, impact on child development, affect general performance, mood and energy levels as well as social relationships and activities (15). Mori et al. examined the wellbeing of caregivers of patients, who were recruited from Australian Rett Syndrome Database, using a family questionnaire (Short Form 12 Health Survey) with 9 years of follow-up. Poorer parental physical wellbeing was found to be related to frequent sleep and behavioral disturbances, living in isolated areas and being a single parent (151). Night waking or severe anxiety impact on both the physical and emotional wellbeing of parents, in agreement with previous research. Sleep problems represent a risk factor not only for parents of RTT patients but also in CDKL5 disorders, autism spectrum disorder and developmental disabilities (151–154). Interventions to improve sleepiness, increase mobility, eye contact and concentration also benefit parents as well as the children themselves (147). Evidence on management of sleep disorders is lacking, as no algorithms are available, the choice of therapy depends on each case (15, 95). Management strategies were investigated by Boban et al., by obtaining data from 364/461 families of patients with RTT registered in the International RTT Phenotype Database. Sleep hygiene strategies were used by 2/3 of the families with a reduction in night waking. The implementation of behavioral strategies including the reduction of environmental stimuli, acting on sleep practice and psychological factors such as mealtimes and exercise, results in a lower impact of the problem on the family, probably due to the benefit that the parents themselves derive from regularizing their habits. In this study 274 patients were not taking any drug treatment; melatonin with clonidine and trazodone were the most frequently used drugs and 21 patients were on polytherapy. The use of drugs corresponded with a higher DIMS (Disorders of initiating and maintaining sleep subscale) score and a higher odds of moderate/major impacts sleep problems. In particular difficulty falling asleep and night waking are the main disorders affecting children undergoing pharmacological treatment (89). Sleep hygiene strategies are the first-line therapy (15, 89). In case of lack of efficacy, the most used drugs include melatonin, dopamine agonist and GABA agonist (89, 155). Studies have shown the effectiveness of melatonin in reducing sleep latency, without affecting other parameters such as nocturnal awakenings. In a randomized placebo-controlled trial in children with neurodevelopmental disorders, melatonin led to an improvement in total sleep time, which was not found in a previous study, and a reduction of sleep latency (15, 114, 156). The side effects of melatonin therapy, which can be seen in the general population, are difficult to assess in patients with neurodevelopmental delay, given the difficulties in communication (89, 157). In the assessment of a patient with RTT, sleep must be considered among the areas of evaluation and its characteristics must be investigated. Also laboratory investigations (ferritin, transferrin, serum iron) should be considered in case of disturbed sleep or restless leg syndrome (86).

## Conclusion

Sleep disorders are common in individuals with intellectual disability and are a chronic problem affecting patients with RTT syndrome (classical RTT and atypical RTT) and their families. Sleep abnormalities as well as seizures and epilepsy are more common in atypical RTT than typical form. Disrupted sleep has detrimental effects on children's development, affects quality of life of patients and their parents. Although studies in humans and animal models have contributed to a broader understanding of causative mechanisms, further research is needed to investigate the molecular, neuronal and non-neuronal pathways underlying sleep disorders and other comorbidities. In addition, actigraphy and polysomnography should be used to further investigate sleep functioning and allow analysis based on objective data. To date, sleep hygiene remains the first-line therapy. Although melatonin has been shown to be effective in improving total sleep time and sleep onset latency, the literature on drug efficacy and safety is poor and drug trials need to be designed.

## Author Contributions

GT and GBD put forward the conception of the review and wrote the manuscript. EM and AV participated in the proposal of the concept and revised the manuscript. GD and PS proposed suggestions for revision. All authors approved the submitted version.

## Conflict of Interest

The authors declare that the research was conducted in the absence of any commercial or financial relationships that could be construed as a potential conflict of interest.

## Publisher's Note

All claims expressed in this article are solely those of the authors and do not necessarily represent those of their affiliated organizations, or those of the publisher, the editors and the reviewers. Any product that may be evaluated in this article, or claim that may be made by its manufacturer, is not guaranteed or endorsed by the publisher.
